# How effective is public health policy in Scotland on vitamin D deficiency during pregnancy?

**DOI:** 10.1017/S1368980023002227

**Published:** 2023-12

**Authors:** Ruth Campbell, Christopher Curran, Jonathan Hayward, Jon Godwin, Susan Johnston, Julie Armstrong, Andrew Collier

**Affiliations:** 1 Department of Public Health, NHS Ayrshire and Arran, Ailsa Hospital, Dalmellington Road, Ayr KA6 6AB, UK; 2 Department of Diabetes and Endocrinology, University Hospital Ayr, NHS Ayrshire and Arran, Dalmellington Road, Ayr KA6 6DX, UK; 3 Nuffield Department of Population Health, University of Oxford, Old Road Campus, Oxford OX3 7LF, UK; 4 Glasgow Royal Infirmary, 84 Castle St, Glasgow G4 0SF, UK; 5 Glasgow Caledonian University, Cowcaddens Road, Glasgow, G4 0BA, UK

**Keywords:** Vitamin D, Supplementation, Pregnancy, Deficiency

## Abstract

**Objective:**

To evaluate the uptake of universal vitamin D supplementation during pregnancy, its effectiveness in preventing vitamin D deficiency and the factors associated with these.

**Design:**

The regional public health organisation in Ayrshire, Scotland has a policy of universal provision of vitamin D supplements (10 µg/d) to all pregnant women for the duration of their pregnancy. Pregnant women in this area were recruited at their 12-week antenatal appointment. Blood samples were collected at the 12-week and 34-week appointments. To account for the seasonal variation, women were recruited in two cohorts: summer and winter. Telephone interviews were conducted at 34 weeks to assess the uptake of vitamin D supplements during pregnancy. Other variables were obtained from medical records.

**Setting:**

The study was conducted in the NHS Ayrshire and Arran Health Board in Scotland.

**Participants:**

612 pregnant women (aged 15–44 years) living in Ayrshire (latitude 55°), Scotland.

**Results:**

Sixty-six percentage took supplementation as recommended. Consumption of supplementation was significantly associated with a higher median serum 25-hydroxyvitamin D concentrations at 34 weeks. Despite this at 34 weeks, 33 % of the summer cohort had insufficient or deficient vitamin D status, while 15 % of the winter cohort had insufficient or deficient status. In multivariable analysis, only adherence and season were independent predictors of vitamin D status.

**Conclusions:**

While supplementation improved and maintained vitamin D status during pregnancy, it was not adequate to ensure all those insufficient at 12 weeks achieved sufficient status at the end of pregnancy.

Vitamin D deficiency has been described in many maternal conditions related to pregnancy and paediatric health; however, its role in their development remains controversial^(1–3)^. Worldwide, vitamin D deficiency is present in more than half of pregnant women and newborns and has been associated with adverse pregnancy outcomes, such as pre-eclampsia, gestational diabetes, fetal growth restriction and preterm labour^(4–6)^. Vitamin D deficiency is therefore considered a major global public health problem in pregnant women of all ages and ethnicities, even in those residing in countries with low latitude and high sun exposure^(7)^.

Vitamin D is produced from a combination of endogenous synthesis (e.g. UVB radiation from sunlight) plus variable dietary sources. On average, approximately 90 % of the body’s vitamin D is provided by endogenous synthesis, while 10 % is provided by diet^(8)^. In recent decades, a substantial body of research investigating the effect of vitamin D supplementation during pregnancy on maternal and child outcomes has been produced. The results of these trials, however, are inconsistent^(2,9)^ and given the marked heterogeneity in the literature regarding the dosage, administration method and stage in pregnancy when the supplementation is administered^(10–15)^ there remains uncertainty over the optimal implementation of vitamin D supplementation during pregnancy. Furthermore, differences in latitude, climate, clothing worn and dietary intake of vitamin D between countries limit comparison^(16)^.

It has been established that vitamin D deficiency is not uncommon in Scottish women with data suggesting 33 % have a serum 25-hydroxyvitamin D (25(OH)D) concentration below 25 nmol/l^(17)^. However, there are little current data on the prevalence of vitamin D deficiency in pregnant women in Scotland and the factors associated with this.

National UK guidance recommending that all pregnant women should take a daily supplement of vitamin D has been in place for a number of years and was reiterated by the Scottish Government in 2017^(18)^. In Scotland where healthcare and public health interventions are organised and provided by regional health board organisations, NHS Ayrshire and Arran has had a policy of provision of vitamin D (10 µg/d), vitamin C (70 mg/d) and folic acid (400 µg/d) supplements to all pregnant women for the duration of their pregnancy since 2012, which has since been expanded to all regional health boards in Scotland. However, the uptake and effect of this universal supplementation of vitamin D provided are unclear.

In this setting, we set out to evaluate the prevalence and factors associated with vitamin D deficiency during pregnancy in this cohort, the uptake and effect of vitamin D supplementation during pregnancy and whether this current policy was effective in preventing vitamin D deficiency in this region.

## Methods

### Study population

To account for the seasonal variation in serum 25(OH)D concentrations, women were recruited at two time points during the year, in both summer and winter. All pregnant women up to 16 weeks gestation who attended for a scan appointment during the two sampling months were invited to participate with no exclusion criteria. In total, 652 women were eligible to take part in the study. Women beyond the 16^th^ week of pregnancy were excluded from the study in addition to participants who consented but subsequently suffered a miscarriage or stillbirth or who moved out of the area.

### Variables

Baseline clinical information including patient demographics, parity, smoking status and BMI was obtained from medical records and patient interview at the first visit. Socio-economic grouping was based on the Scottish Index of Multiple Deprivation (SIMD) 2013 and was derived using patient postcodes. SIMD is a relative measure across 6976 data zones in Scotland, measuring the extent to which a geographical area is deprived across seven domains: income, employment, education, education, health, access to services, crime and housing^(19)^. SIMD quintiles ranged from 1 (most deprived) to 5 (least deprived). Women at their 12 week scan appointment in August 2014 were called the summer cohort, and those invited to take part in February 2015 were called the winter cohort.

### Measurement of serum 25-hydroxyvitamin D

Blood samples for the measurement of serum 25(OH)D concentrations were collected at the 12-week and 34-week appointments. Vitamin D was analysed using the liquid chromatography-tandem mass spectrometry to measure 25-hydroxyvitamin D_2_ (25(OH)D2) and 25-hydroxyvitamin D_3_ (25(OH)D3) concentrations in each sample^(20)^. The laboratory involved used calibrators traceable to NIST 972a reference material and is therefore comparable to other research using the CRM-aligned liquid chromatography-tandem mass spectrometry methods. The intra- and inter-assay coefficients of variance were < 10 % over the concentration range of 23–120 nmol/l for 25(OH)D3 and 18–70 nmol/l for 25(OH)D2. The lowest detectable concentration for 25(OH)D2 and 25(OH)D3 was 8 nmol/l. Undetectable concentrations of less than 8 nmol/l were recorded as 7·99 nmol/l. Total serum 25(OH)D was derived from the sum of 25(OH)D2 and 25(OH)D3. Vitamin D status was defined similar to previous studies including those on pregnant women by categorising total serum 25(OH)D into the following ranges: deficiency < 25 nmol/l; insufficiency 25–50 nmol/l; sufficiency 51–75 nmol/l and optimal > 75 nmol/l^(21,22)^.

### Vitamin D supplementation and adherence

The regional public health organisation has a policy of universal provision of vitamin D supplements (10 µg/d) to all pregnant women for the duration of their pregnancy in the form of a multivitamin tablet containing 10 µg of vitamin D, 400 µg of folic acid and 70 mg of vitamin C. Women are given four bottles of fifty-six tablets at the first ultrasound scan appointment, which usually takes place around the 12th week of pregnancy. Additionally, they are informed at the booking appointment about the importance of taking a daily vitamin D supplement.

Telephone interviews were conducted around 35–38 weeks gestation to assess the uptake of vitamin D supplements during pregnancy. A structured interview schedule (see online supplementary material, Supplemental file 1) was used, which included questions on the duration, dose and frequency of consumption of the vitamin supplements provided. Participants who reported that they did not take the vitamin supplements issued were asked if they took an alternative vitamin supplement. Those who did take an alternative were subsequently asked the same questions relating to duration, dose and frequency of consumption.

For women to be regarded as adherent to the vitamin D supplementation advice, they had to meet the following criteria: they started taking the provided vitamin supplements (or an alternative containing 10 µg/d of vitamin D) when they were first issued by their midwife, they were still taking these at the time of the interview (at 35–38 weeks) plus they took one tablet per day greater than 4 d per week. If women did not meet all these criteria, they were classed as not having taken a vitamin D supplement as recommended.

### Statistical analysis

Fisher’s exact test was used to compare two categorical dichotomous variables. A trend test (or linear by linear association) was used to compare an ordinal variable by two characteristics. Univariate and multivariate logistic regression analysis was used to test the factors associated with vitamin D status. Variables with *P* ≤ 0·2 in the univariate logistic regression analysis were selected for entry into the multivariate logistic regression model. The following independent variables were included in the multivariable model: age, SIMD, BMI, smoking status, season of collection of the 34-week blood sample and the uptake of vitamin D supplements. A *P* value of < 0·05 was considered statistically significant. Bonferroni correction was applied where there were multiple *P* values. SPSS software version 22 IBM SPSS Statistics was used for all statistical analyses.

## Results

### Participants and descriptive data

**Fig 1:**
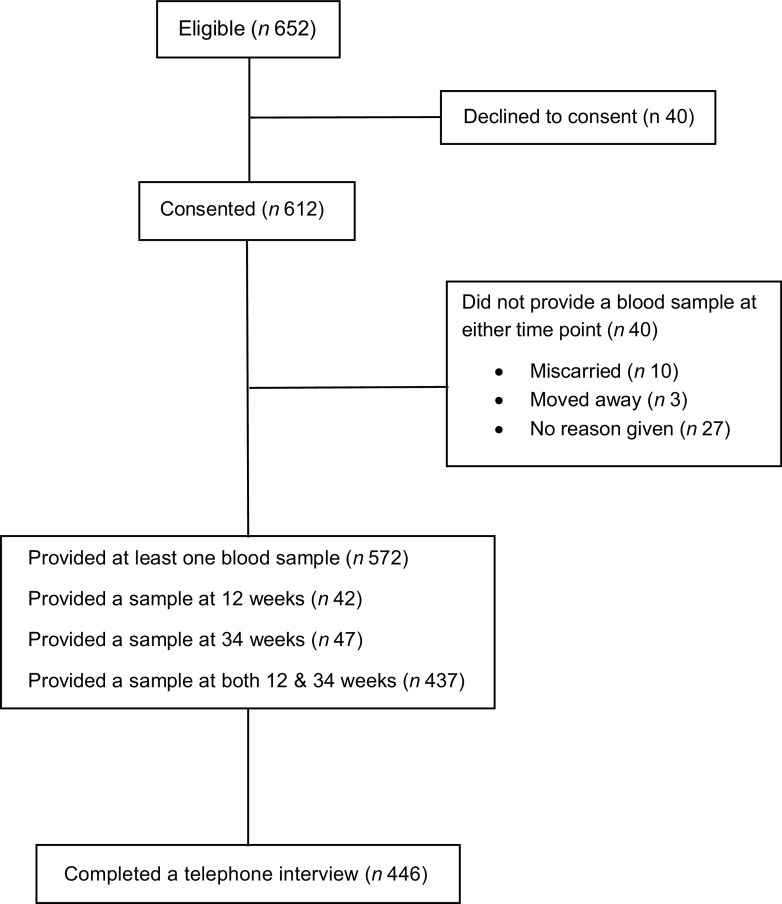
Flow diagram

In total, 652 pregnant women were eligible to take part in the study. Six hundred and twelve women gave consent to their inclusion in the study at their 12-week scan appointment. Five and seventy-two women provided at least one blood sample. Five and forty-two women (89 %) provided a blood sample at 12 weeks, 467 women (76 %) provided a blood sample at 34 weeks and 437 women (71 %) provided a blood sample at both time points. Of 446 women who took part in an interview, 414 provided a blood sample at 12 and 34 weeks (Fig. 1). Forty women did not provide a blood sample at either time point due to suffering a miscarriage, moving out of area or for a reason not given. There were no differences in the characteristics of women who provided a blood sample at 12 or 34 weeks and those who consented but did not provide a sample at either point.

The demographic characteristics of all participants who provided at least one blood sample are shown in Table 1. The median age was 28 years and ranged from 15 to 44 years. In this cohort, 60·2 % lived in an area classified within the two most deprived SIMD quintiles, while only 10 % of all participants lived in area classified within the least deprived quintile. Median BMI (inter-quartile range (IQR)) was 25 (8), and 53 % of all women were either overweight or obese and more than half of all participants (57 %) were expecting their second or subsequent child. There were 548 (95·8 %) women with an ethnic origin of UK White with only twenty-four women (4 %) of an ethnic origin other than this.

**Table 1:** Demographic characteristics of all participants who provided at least one blood sample

Variable	(*n* 572)	
	*n*	%
Age (years)		
< 20	29	5·1
20–29	292	51·0
30 and over	251	43·9
SIMD		
1 (most deprived)	171	29·9
2	170	29·7
3	92	16·1
4	78	13·6
5 (least deprived)	55	9·6
Unavailable	6	1·1
BMI (kg/m^2^)		
< 18·5	16	2·8
18·5–25	252	44·1
25–29·9	157	27·4
≥ 30	147	25·7
Smoking status		
Non-smoker	459	80·2
Smoker	113	19·8
Parity		
Primiparous	248	43·4
Multiparous	324	56·6
Ethnicity		
UK White	548	95·8
African/African Caribbean (Black)	1	0·2
South Asia (Asian)	5	0·9
South East Asia (Asian)	4	0·7
Other non-European	1	0·2
Southern and other European (White)	5	0·9
Northern European (White)	8	1·4

SIMD, Scottish Index of Multiple Deprivation quintile was missing for six participants. *n* = total number of patients who provided at least one blood sample.

### Vitamin D status

The median total serum 25(OH)D concentration at 12 weeks (*n* 542) was 61 nmol/l (IQR 40) and ranged from < 8 to 144 nmol/l. The median total serum 25(OH)D concentration at 34 weeks (*n* 467) was 81 nmol/l (IQR 51) and ranged from < 8 to 205 nmol/l.

Among women who took a vitamin D supplement as recommended, median serum 25(OH)D concentration at 34 weeks was significantly higher than those who did not (89 nmol/l (IQR 41) *v*. 57 nmol/l (IQR 56), respectively, *P* < 0·001).

The proportion of women in each vitamin D category at 12 and 34 weeks is shown in Table 2. Of the 542 women who provided a blood sample at 12 weeks, 36·4 % had a serum 25(OH)D status in the deficient or insufficient category (18·5 % in the summer cohort compared with 49·2 % in the winter cohort (*P* < 0·001)). Of the 467 women who provided a blood sample at 34 weeks, 22·7 % were found to be deficient or insufficient in serum 25(OH)D overall (33·4 % in the summer cohort compared with 15·5 % in the winter cohort (*P* < 0·001)).

**Table 2:** Vitamin D status at 12 and 34 weeks by season

	All				Summer cohort*				Winter cohort†			
Vitamin D category	12 weeks		34 weeks		12 weeks (summer)		34 weeks (winter)		12 weeks (winter)		34 weeks (summer)	
	*n* 542	%	*n* 467	%	*n* 227	%	*n* 189	%	*n* 315	%	*n* 278	%
Deficient												
< 25 nmol/l	41	7·6	31	6·6	4	1·8	26	13·8	37	11·7	5	1·8
Insufficient												
25–50 nmol/l	156	28·8	75	16·1	38	16·7	37	19·6	118	37·5	38	13·7
Sufficient												
> 50–75 nmol/l	182	33·6	98	21·0	82	36·1	47	24·9	100	31·7	51	18·3
Optimal												
> 75 nmol/l	163	30·1	263	56·3	103	45·4	79	41·8	60	19·0	184	66·2

*n* = number of patients providing a blood sample at that time point by season.

* Summer cohort recruited at 12 weeks gestation in August 2014.

† Winter cohort recruited at 12 weeks gestation in February 2015.

### Factors associated with vitamin D deficiency at 34 weeks

Table 3 shows the unadjusted OR and 95 % CI for variables associated with vitamin D status at 34 weeks. Parity was not significantly associated with serum 25(OH)D at 34 weeks. Only two variables were significantly associated with serum 25(OH)D insufficiency at 34 weeks: season of collection of 34 week blood sample and uptake of vitamin D supplements as recommended.

**Table 3:** OR (95 % CI) for deficiency or insufficiency of vitamin D at 34 weeks

Variable	*n*	Unadjusted OR	95 % CI	*P*	*n*	Adjusted OR	95 % CI	*P*
Age (years)								
< 20	19	Ref	–	–	17	Ref	–	–
20–29	233	0·91	0·33–2·50	0·86	213	1·62	0·45–5·88	0·46
> 30	215	0·37	0·13–1·03	0·06	205	0·91	0·24–3·46	0·89
SIMD								
1	129	2·05	0·87–4·82	0·01	118	1·52	0·51–4·53	0·45
2	149	1·16	0·49–2·76	0·73	140	1·01	0·34–2·99	0·98
3	76	1·05	0·40–2·72	0·93	73	0·93	0·29–2·99	0·9
4	65	0·68	0·24–1·94	0·47	65	0·84	0·24–2·95	0·78
5 (least deprived)	42	Ref	–	–	39	Ref	–	–
BMI (kg/m^2^)								
< 25	212	Ref	–	–	192	Ref	–	–
25–29·9	136	1·07	0·63–1·81	0·82	130	1·1	0·58–2·09	0·78
> 30	119	1·57	0·94–2·64	0·09	113	1·44	0·76–2·72	0·27
Smoking status								
Non-smoker	391	Ref	–	–	370	Ref	–	–
Smoker	76	1·88	1·10–3·21	0·02	65	1·01	0·49–2·07	0·98
Season of 34 week blood collection								
Summer group	189	Ref	–	–	177	Ref	–	–
Winter group	278	0·37	0·24–0·57	< 0·001	258	0·22	0·13–0·39	< 0·001
Vitamin supplementation								
Supplements taken	293	Ref	–	–	287	Ref	–	–
Supplements not taken	148	6·44	3·93–10·56	< 0·001	148	7·72	4·36–13·66	< 0·001

SIMD, Scottish Index of Multiple Deprivation quintile missing for six participants.

### Uptake of supplementation

Four hundred and fourteen women interviewed (93 %) reported that they took some form of the provided vitamin D supplementation at some point during their pregnancy. Of these, 295 (66 %) took a vitamin D supplement as recommended. There was a significant difference in the uptake of vitamin D supplements with age, SIMD and smoking status (Table 4). Older women aged 30 or over, those living in the least deprived areas and non-smokers were more likely to have taken vitamin D supplements as recommended compared with younger women, those living in the most deprived areas and those who smoked (*P* < 0·001; *P* = 0·001; *P* = 0·001, respectively). BMI and parity were not associated with the uptake of vitamin D supplements.

**Table 4:** Uptake of vitamin supplements by age, SIMD, BMI, smoking status and parity

	Participants who took supplements taken as recommended		Participants who did not take vitamin supplements as recommended		
	(*n* 295)		(*n* 151)		
	*n*	%	*n*	%	*P*
Age (years)					< 0·001
< 20	6	35·3	11	64·7	
20–29	129	58·9	90	41·1	
30 and above	160	76·2	50	23·8	
SIMD					0·001
1 (most deprived)	71	59·2	49	40·8	
2	85	59·9	57	40·1	
3	51	68·9	23	31·1	
4	52	80·0	13	20·0	
5 (least deprived)	30	76·9	9	23·1	
BMI (kg/m^2^)					0·3
< 25	135	67·8	64	32·2	
25–29·9	88	67·7	42	32·2	
30 and above	72	61·5	45	38·5	
Smoking status					0·001
Non-smoker	262	69·5	115	30·5	
Smoker	33	47·8	36	52·2	
Parity					0·23
Primiparous	132	69·5	58	30·5	
Multiparous	163	63·7	93	36·3	

SIMD, Scottish Index of Multiple Deprivation quintile missing for six participants. Total *n* = number of patients who took part in an interview (446). Difference in the uptake of vitamin supplements by age, SIMD and BMI was assessed using trend test (linear by linear association). Difference in the uptake of vitamin supplements by smoking status and parity was assessed using Fisher’s exact test.

## Discussion

This study prospectively assessed the vitamin D status of a cohort of pregnant women living in Ayrshire and Arran and the effect of universally provided vitamin D supplementation on their vitamin D status. A high uptake of supplementation was present with 93 % taking some form of vitamin D supplementation with 66 % following the vitamin D supplement guidance. The consumption of vitamin D supplements was significantly associated with higher median serum 25(OH)D concentrations at 34 weeks regardless of season. Despite this, at 34 weeks 33 % of the summer cohort had insufficient or deficient vitamin D status, while 15 % of the winter cohort had insufficient or deficient status. Interestingly, although univariate analysis showed older women (> 30 years), non-smokers and women living in least deprived areas were more likely to take the vitamin D supplements, multi-variable analysis revealed that only adherence and season were independent predictors of vitamin D status. In short, despite the consumption of the recommended dose, a significant proportion of both cohorts had insufficient serum 25(OH)D concentration in the late stages of pregnancy.

Our findings of higher median serum 25(OH)D concentrations in association with vitamin D supplementation adds to previous research in this area. It has been demonstrated that pregnant women on supplemented vitamin D had significantly higher serum 25(OH)D concentrations compared with controls^(2)^. This may be of clinical significance given that higher serum 25(OH)D concentrations have been associated with a reduced risk of adverse pregnancy outcomes, including pre-eclampsia, gestational diabetes and pre-term birth^(3)^.

The strengths of this study are in its prospective evaluation of vitamin D status from a large cohort of pregnant women throughout their pregnancy. Participants were recruited from all antenatal clinics in the region, and there were limited exclusion criteria suggesting the study cohort was representative of the local population. Additionally, detailed assessment of uptake of supplementation was possible from the telephone interview conducted after 34 weeks. Importantly, by recruiting cohorts during both summer and winter we were able to adjust for seasonal variation of serum 25(OH)D concentrations. We believe this is the first study in Scotland to measure the impact of public health policy providing universal vitamin D supplementation on the vitamin D status of women in early and late pregnancy. Promisingly, we recorded a high uptake of supplementation suggesting current policy is a pragmatic and effective means of enabling women to access and consume vitamin D supplements during pregnancy. In addition, our results corroborate previous research done in the UK^(13)^ demonstrating higher compliance with supplementation and delivery in summer are independent predictors of a greater serum 25(OH)D concentrations in late pregnancy.

The study however had several limitations that should be considered when interpreting the results. Principally, the relatively modest dose employed in this study prevents firm conclusions being drawn on the efficacy of supplementation in preventing vitamin D deficiency at the end of pregnancy. Given the well-established high prevalence of vitamin D deficiency in pregnancy^(23,24)^ and considering that the recommended dose to treat non-pregnant individuals with vitamin D deficiency is ten times that employed here^(21,22)^, it is possible this dose is too small to prevent deficiency in all women at the end of gestation. This hypothesis is supported by the evidence from research in the UK^(13)^ where 25 % of mothers delivering in winter had a serum 25(OH)D concentrations of less than 50 nmol/l despite supplementation with 25 µg/d of vitamin D. Furthermore, randomised controlled trials of supplementation with vitamin D to pregnant women in America and the UAE demonstrate doses of 50 and 100 µg/d were safe and superior to 10 µg/d in achieving a significantly higher mean serum 25(OH)D concentration at delivery^(10,11)^. This could explain in part the large number of women with a serum 25(OH)D concentration in the insufficient range observed at the latter stages of pregnancy in our study.

Additionally, the measurement of dietary intake of vitamin D was not included. Previous studies among pregnant women have found mean dietary intake to be 3·6–3·7 µg per day^(21,25)^. Accounting for this, together with the difficulties in accurately measuring dietary intake of vitamin D, an assumption was made at the study design phase that women in the cohort would have a dietary intake within these ranges. Sun exposure is also a key determinant of vitamin D status; however, data were not collected on time spent outdoors or holidays taken abroad; therefore, sun exposure was not assessed. This is a likely important confounder and particularly relevant when consideration is given to the advice from the Scottish Government on limiting sunlight exposure and the widespread use of sunscreen in response to concerns regarding skin cancer^(26,27)^. Furthermore, the study sample was relatively homogenous in terms of ethnicity with >95 % being Caucasian; therefore, the number of women from ethnic groups included in the sample was insufficient to assess the effect of ethnicity on vitamin D status. This finding however simply reflects the lower number of ethnic minority groups in Ayrshire and Arran compared with the Scottish average.

After multivariate regression, only season and the uptake of vitamin D supplementation proved independent predictors of vitamin D status in our cohort of women in Scotland. There is conflicting evidence in the literature on the predictors of vitamin D status in pregnant women likely due to the heterogeneous nature of both the populations studied and design of the mainly observational studies. For example, research in Shanghai, China did demonstrate associations between vitamin D deficiency in pregnancy and age over 30, parity over 2, increased BMI and hyperlipidaemia^(28)^. However, this study excluded pregnant women who used vitamin D supplementation, thus limiting comparison to our work. Similarly, a large Danish study demonstrated vitamin D deficiency was associated with winter season, increased pre-pregnancy BMI, smoking and multiparity^(29)^.

Conversely, however, a prospective observational study of 220 women in Korea found no association between characteristics such as age, smoking, parity and BMI and vitamin D status^(30)^. Both the Danish and Korean research collected no data on vitamin D supplementation, thus limiting interpretation and further comparison with our results. Other differences between these studies and ours may be due to unmeasured confounders relating to the demographic and cultural differences present such as ethnicity, diet and sun exposure.

The Maternal Vitamin D Osteoporosis Study was a randomised controlled trial of vitamin D supplementation compared with placebo in pregnant women in the UK^(13)^. In those women randomised to cholecalciferol, lower compliance with supplementation and delivery in the winter *v*. the summer were independently associated with lower 25(OH)D concentration at 34 weeks gestation. Our results confirm these are likely two of the strongest predictors of vitamin D status in pregnant women in the UK.

A further consideration is the lack of an international consensus regarding the best method for measuring vitamin D and the cut-off points for vitamin D insufficiency and sufficiency^(31)^. In this study, 25(OH)D2 and 25(OH)D3 were measured using the liquid chromatography-tandem mass spectrometry at the regional laboratory in Glasgow Royal Infirmary, a member of the Vitamin D External Quality Assessment Scheme (DEQAS). This laboratory uses calibrators traceable to NIST 972a reference material; this method is comparable to other studies using the certified reference material-aligned liquid chromatography-tandem mass spectrometry methods. Despite the presumption that this technique is the gold standard, variability of assays is widely recognised, thus limiting comparison with other studies which use methods that are not traceable to reference method procedures.

Finally, adherence to vitamin D supplementation was assessed during telephone interviews. The interview was structured and patients were asked to clarify their adherence; however, studies have shown that this method of assessment can lead to an over-reporting of adherence compared with more accurate metrics such as pill counts^(32)^.

The role of vitamin D supplementation in pregnancy remains controversial given mixed results of current randomised control trial evidence^(33–35)^. A 2018 review suggested that a replete serum 25(OH)D concentration of vitamin D is associated with a greater chance of reproductive treatment success^(36)^. Furthermore, studies have suggested a link between vitamin D deficiency and sporadic spontaneous abortion^(37)^ and other poor perinatal outcomes, including gestational diabetes, pre-eclampsia, pre-term birth and low birth weights^(4,5)^. There is however suggestion that the effect of vitamin D replacement is not clinically important with other research suggesting that vitamin D deficiency may be an indicator for poor health status, comorbidities or perhaps an acute phase reactant^(38,39)^. Unfortunately, assessing the effect of vitamin D supplementation on clinical endpoints was beyond the scope of this study.

### Conclusion

We have demonstrated that universal supplementation with vitamin D is a feasible and practical approach to increasing the serum 25(OH)D concentrations in pregnant women with a majority of women taking the provided supplements as recommended. However, supplementation as implemented here was not sufficient to ensure all women had sufficient serum 25(OH)D concentrations at the end of pregnancy. Given the uncertainty surrounding the benefit of vitamin D supplementation during pregnancy on clinical outcomes of mother and infant, further research is clearly necessary. We would suggest this could be conducted using a larger dose of vitamin D, with longer-term follow-up and assessment of clinical endpoints, including maternal conditions related to pregnancy (such as pre-eclampsia and gestational diabetes) and paediatric health (such as preterm birth, birth weight and respiratory conditions).

## Supporting information

Campbell et al. supplementary material Campbell et al. supplementary material

## Supplementary material

For supplementary material accompanying this paper visit https://doi.org/10.1017/S1368980023002227
