# Supplementary material for: How effective is public health policy in Scotland on vitamin D deficiency during pregnancy?
Source: Public Health Nutr. 2023 Oct 26;26(12):3311–9. doi: 10.1017/S1368980023002227 (PMC10755384; doi:10.1017/S1368980023002227)
Supplement: Campbell et al. supplementary material [file S1368980023002227sup001.docx]

Supplementary file 1: Interview schedule

**Vitamin D in Pregnancy Study**

Participant’s name: ................................................................................................

Participant number: ...............................................................................................

Date(s) of first & any subsequent contact: ............................................................

Date of telephone interview: ..................................................................................

1. At your booking appointment around 12 weeks, did your midwife advise you to take a supplement containing vitamin D?

Yes

No

Don’t remember

2. Were you given 4 bottles of Healthy Start vitamins at your booking appointment?

Yes

No

Don’t remember

3. Did you take the Healthy Start vitamins?

Yes If yes, go to questions 4 – 8

No If no, go to question 9

4. When did you start taking the Healthy Start vitamins?

Between 6 – 12 weeks

Between 13 – 26 weeks

After 26 weeks

Don’t remember

5. Are you still taking the Healthy Start vitamins now?

Yes If yes, go to question 7

No If no, go to question 6

6. When did you stop taking the Healthy Start vitamins?

Between 13 – 26 weeks

Between 27 – 32 weeks

After 32 weeks

Don’t remember

7. How many Healthy Start vitamins did/do you take?

One tablet per day

Two tablets per day

More than two tablets per day

Don’t remember

8. How often did/do you take the Healthy Start vitamins?

Every day

5-6 days of the week

3-4 days of the week

1-2 days of the week

Less than once per week

End of interview, no further questions. Thank participant for taking part.

Questions 9 – 13 only for those who responded ‘No’ to question 3

9. Did you take another vitamin supplement containing vitamin D?

Yes If yes, go to Q10

No If no, end of interview

10. When did you start taking the supplement?

Between 6 – 12 weeks

Between 13 – 26 weeks

After 26 weeks

Don’t remember

11. Are you still taking the supplement now?

Yes If yes, go to question 13

No If no, go to question 12

12. When did you stop taking the vitamins?

Between 13 – 26 weeks

Between 27 – 32 weeks

After 32 weeks

Don’t remember

13. What is the name of the supplement you took/are taking?

..................................................................................................................................

14. How much of the supplement did/do you take?

One tablet per day

Two tablets per day

More than two tablets per day

Don’t remember

15. How often did/do you take the supplement?

Every day

5-6 days of the week

3-4 days of the week

1-2 days of the week

Less than once per week

End of interview. Thank participant for taking part.
